# Synthesis of 1,4-Disubstituted Mono and Bis-triazolocarbo-acyclonucleoside Analogues of 9-(4-Hydroxybutyl)guanine by Cu(I)-Catalyzed Click Azide-Alkyne Cycloaddition

**DOI:** 10.3390/molecules17010179

**Published:** 2011-12-27

**Authors:** Jamal Krim, Moha Taourirte, Joachim W. Engels

**Affiliations:** 1 Laboratoire de Chimie Bioorganique et Macromoléculaire, Faculté des Sciences et Techniques - Guéliz, 40000, Marrakech, Maroc; 2 Institut für Organische Chemie und Chemische Biologie, J.W. Goethe Universität, Max-von-Laue Str. 7, 60438 Frankfurt am Main, Germany

**Keywords:** 1,3-dipolar cycloaddition, 1,2,3 triazole, 1,2,3-bis-triazoles, click azide-alkyne, microwave-assisted synthesis, antibacterial activities

## Abstract

A series of novel mono-1,2,3-triazole and bis-1,2,3-triazole acyclonucleoside analogues of 9-(4-hydroxybutyl)guanine was prepared *via* copper(I)-catalyzed 1,3-dipolar cycloaddition of *N*-9 propargylpurine, *N*-1-propargylpyrimidines/as-triazine with the azido-pseudo-sugar 4-azidobutylacetate under solvent-free microwave conditions, followed by treatment with K_2_CO_3_/MeOH, or NH_3_/MeOH. All compounds studied in this work were screened for their antiviral activities [against human rhinovirus (HRV) and hepatitis C virus (HCV)] and antibacterial activities against a series of Gram positive and negative bacteria.

## 1. Introduction

**Figure 1:**
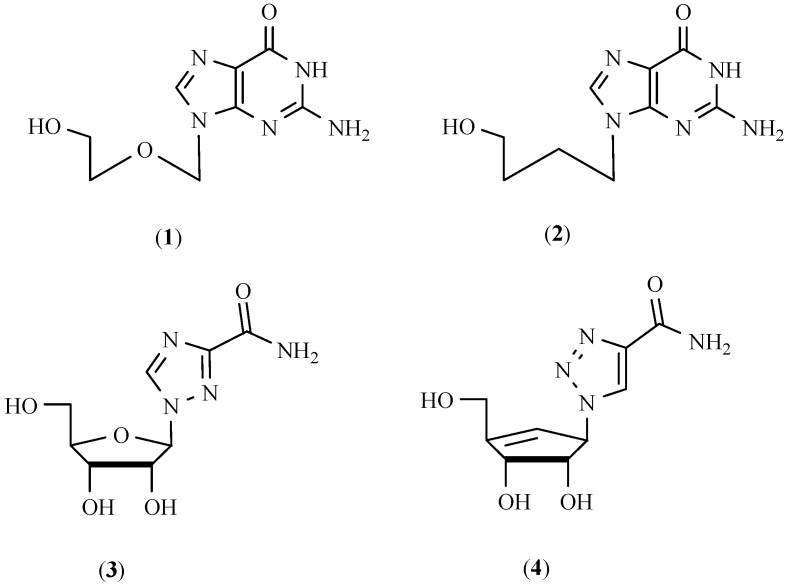
Examples of drugs effective in chemotherapy.

For several years, there has been an intensive search for drugs effective in chemotherapy of viral diseases like AIDS, herpes simplex, Hepatitis C and cytomegaloviruses [1,2,3,4,5]. Most of these drugs are analogues of naturally occurring nucleosides [6]. A series of nucleoside analogues were synthesised in which the cyclic carbohydrate moiety was replaced by an acyclic side chain [7,8,9,10,11,12]. The biological activities of acyclonucleosides, after the discovery of acyclovir [9-((2-hydroxyethoxy) methyl)guanine ACV (Zovirax)] (**1**, Figure 1), have led to the synthesis of a diversity of structures. Many variations were tested in order to enhance biological activity and selectivity, or to lower toxicity [13,14,15,16,17,18]. Among them HBG [9-(4-hydroxybutyl)guanine] (**2**, Figure 1) presented good activity against HSV-1 and HSV-2.

On the other hand, for antiviral agents, triazolonucleosides and acyclonucleosides have attracted much attention. Ribavirin (**3**, Figure 1), whose nucleobase consists of an unnatural triazole moiety, was the first synthetic nucleoside to show a broad spectrum of antiviral activities against many RNA and DNA viruses [19]. Furthermore, nucleosides with unnatural triazole nucleobases are generally resistant to nucleos(t)ide metabolizing enzymes, and this may lead to better *in vivo* stability and efficiency. Because of their broad application as pharmaceuticals like antibacterial or antiviral agents, a great number of 1,2,3-triazole derivatives have been reported as potent antiviral, antimicrobial or antiproliferative agents [20]. Also the synthesis and biological evaluation of carbonucleosides (substances in which the anomeric oxygen of the furanose ring is replaced by a methylene group), having a 1,2,3-triazole ring as a nucleobase (e.g., **4**, Figure 1) have been reported. Until now, very few efforts were made on appending aromatic systems to triazole nucleosides. We expect that these extended aromatic systems may offer advantageous binding properties to the corresponding biological targets via larger aromatic systems.

## 2. Results and Discussion

Different synthetic methods have been developed for the construction of triazole frameworks. These compounds are typically prepared by thermal cycloaddition of azides and alkynes [21,22]. Two problems are, however, encountered in this transformation: (1) reactivity of the substrates, either alkynes or azides require activation by an electron withdrawing group, otherwise, the reaction must be carried out at higher temperatures; (2) the regioselectivity of the products, as for unsymmetrical alkynes, a mixture of regioisomers is obtained in most cases. Since Sharpless reported copper(I) catalysis for regioselective cycloaddition of terminal alkynes and azides to yield exclusively 1,4-disubstituted-1,2,3-triazoles, many groups have reported their results employing different kinds of Cu(I) salts as catalyst [23,24,25,26,27,28,29,30,31,32,33,34,35]. In addition, microwave irradiation has become a powerful synthetic tool for rapid synthesis of a variety of biologically active compounds. Its use to is to enhance the rates of classical organic reactions.

In the light of these findings and in continuation of our previous investigation [34], we considered the synthesis of new 1,2,3-triazole and bis-1,2,3-triazole acyclonucleosides. They carry either a purine, pyrimidine or as-triazine moiety as nucleobase appended to 1,2,3-triazole. They can be regarded as analogues of 9-(4-hydroxybutyl)guanine (HBG). We went further to combine nucleobase and triazole rings with an acyclic side-chain developed bistriazolyl acyclonucleosides, and determined their *in vitro* antiviral and antibacterial activities.

### 2.1. Chemistry

**Scheme 1:**
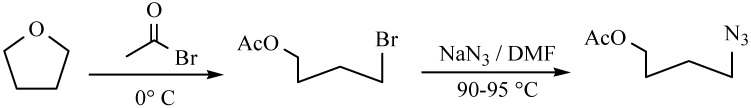
Synthesis of the azidobutylacetate **7** from bromobutylacetate **6**.

The starting material 4-azidobutylacetate (**7**) was prepared according to the literature [34,36] from 4-bromobutylacetate (**6**) and sodium azide at 90–95 °C for 4 h (Scheme 1).

**Scheme 2:**
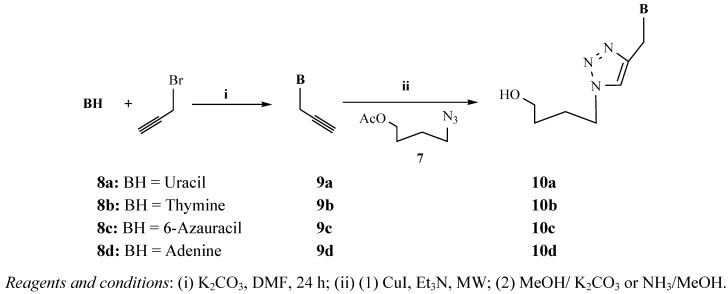
Preparation of mono-triazolo-carboacyclonucleosides **10a–d**.

The second step of the synthesis was the preparation of monopropargylated nucleobases. For this, uracil, thymine, 6-azauracil and adenine were used as starting materials that were treated with propargylbromide in the presence of K_2_CO_3_. All reactions were carried out in DMF, as it is an excellent solvent for dissolving nucleobases [34] (Scheme 2). The pyrimidine and as-triazine derivatives were exclusively alkylated at the *N*-1 position, (**9a**–**c**), and the purine in *N*-9 position, (**9d**) as confirmed by ^1^H-NMR and ^13^C-NMR spectra.

The terminal triple bonds of propargylated nucleobases were ligated to the azide residue of the pseudosugar using copper(I)-catalyzed 1,3-dipolar cycloaddition and Et_3_N under microwave-assisted reaction without solvent [34] (Scheme 2) leading to the 1,4-disubstituted regioisomer in a quantitative yield unlike before [22] and a reaction time of one minute (Table 1). We intimately mixed the azide, acetylenic derivative and copper(I)-iodide prior to microwave irradiation. This fast and efficient method was in all tested cases superior in yield and handling to running the reaction in solution [34].

**Table 1:** Structures of the starting azides, alkynes and corresponding products.

Entry	Azide	Alkyne	Reaction time	Product ^a^	Yields(% ^b^)
**10a**	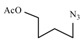	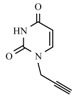	1 min	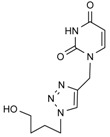	91
**10b**	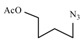	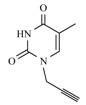	1 min	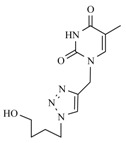	92
**10c**	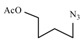	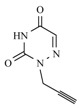	1 min	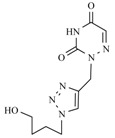	90
**10d**	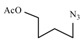	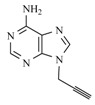	1 min	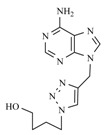	89
**12a**	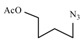	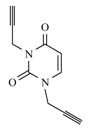	1 min	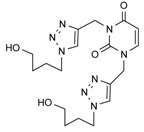	90
**12b**	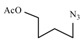	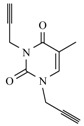	1 min	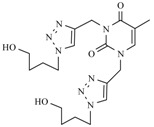	92
**12c**	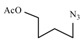	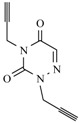	1 min	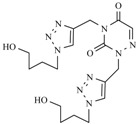	91

^a^ All products were characterized by ^1^H-NMR, ^13^C-NMR, and mass spectrometry; ^b^ Yields of isolated products after deacetylation.

A common feature of many acyclic nucleoside analogues showing biological activity, including HBG, is the presence of a primary alcoholic group. This function and the nucleic acid base are essential for their biological activity. For this purpose the deprotected products were obtained in good yields by treatment with NH_3_/methanol or K_2_CO_3_/methanol.

**Scheme 3:**
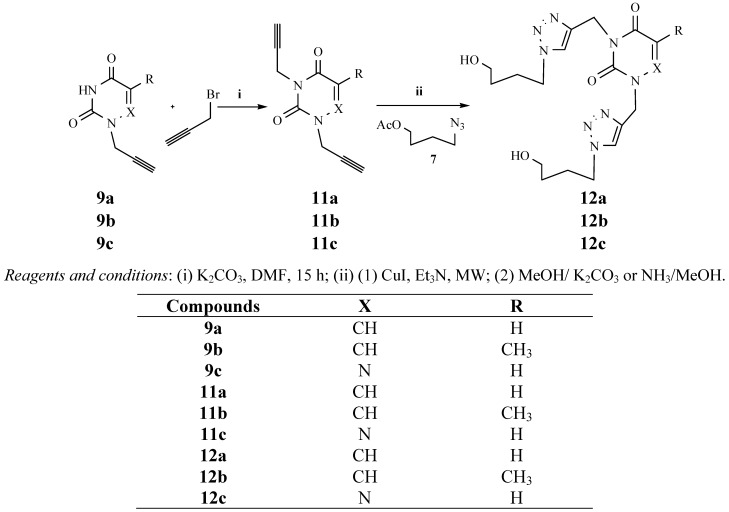
Preparation of bis-triazolo-carboacyclonucleosides **12a–c**.

To extend the general applicability of the microwave assisted click reaction for the synthesis of triazole acyclonucleosides we included other alkinyl derivatives, as outlined in Scheme 2. Analogously to the preparation of *N*-1-propargylated pyrimidines/as-triazine, the *N*-1, *N*-3-bis-propargylated pyrimidines/as-triazines were prepared from *N*-1-propargylated uracil, thymine and 6-azauracil (Scheme 3), (Yields 80–85%). The bis-propargylated pyrimidines/as-triazines were converted into the bis-triazole acyclonucleosides using the same reaction conditions in an almost quantitative yield (Table 1).

The structure of all compounds was confirmed on the basis of ^1^H-, ^13^C-NMR spectra and mass spectra. Formation of 1,4-disubstituted triazoles was unequivocally established by the characteristic chemical shift values of the triazolyl proton (5-CH) at δ = 7.91–8.10 ppm. The triazole ring formation can also be identified from the 13C-spectra with the new signals of the olefinic C-atoms of the 1,2,3-triazole moiety at (δ (C5) = 122.94–123.39 ppm) and (δ(C4) =141.48–145.44 ppm).

### 2.2. Biological Results

#### 2.2.1. Antibacterial Activity

The antibacterial activity of all the synthesized compounds **10a**–**d** and **12a**–**c** were examined against different Gram-positive *Staphylococcus aureus* (ATCC 13709 *in vivo*, ATCC 25923, oxford and MRSA *in vivo*), *Enterococcus faecalis* (ATCC 29212 VanS), *Enterococcus faecium* (VanA), *Streptococcus pneumoniae* (VanA, ATCC49619, PenR and Blood effect), and Gram-negative *Haemophilus influenzae* (ATCC 31517 MMSA), *Escherichia coli* (ATCC 25922) *Pseudomonas aeruginosa* (ATCC 27853). We measured the minimum inhibitory concentration (MIC) values, which are defined as the lowest concentration of an antimicrobial that visibly inhibits the growth of the bacteria after an overnight incubation [37]. Ciprofloxacin and linezolid were used as standard drugs for comparison (Table 2). As shown in Table 2, no antibacterial activities against Gram-positive and Gram-negative bacteria were noted. All compounds showed antibacterial activity with a range of the MICs higher than 64 µg/mL.

**Table 2:** Minimum inhibitory concentration (MIC) in μg/mL of medium.

Strains			Phenotype	Cipro	Lin	10a	10b	10c	10d	12a	12b	12c
**1**	*S. aureus*	Sa1	ATCC13709 *in vivo*	0.12	1	>64	>64	>64	>64	>64	>64	>64
**2**		Sa26	ATCC25923	0.25	1	>64	>64	>64	>64	>64	>64	>64
**3**		Sa26 + 10% Human serum	Serum effect	0.25	1	>64	>64	>64	>64	>64	>64	>64
**4**		Sa26 + 50% Human serum	Serum effect	0.5	2	>64	>64	>64	>64	>64	>64	>64
**5**		Sa4	Oxford	0.12	1	>64	>64	>64	>64	>64	>64	>64
**6**		Sa2	MRSA, *in vivo*	8	1	>64	>64	>64	>64	>64	>64	>64
**7**	*E. faecalis*	Ecalis1	ATCC29212 VanS	0,5	2	>64	>64	>64	>64	>64	>64	>64
**8**	*E. faecium*	Ecium1	VanA	16	0.5	>64	>64	>64	>64	>64	>64	>64
**9**	*S. pneumoniae*	Pn1	ATCC49619	1	1	>64	>64	>64	>64	>64	>64	>64
**10**		Pn9	PenR	0.5	0.5	>64	>64	>64	>64	>64	>64	>64
**11**		Pn9+2.5% blood	Blood effect	0.5	0.25	>64	>64	>64	>64	>64	>64	>64
**12**	*H. influenzae*	Hi3	ATCC 31517 MMSA	≤0.03	16	>64	>64	>64	>64	>64	>64	>64
**14**	*E. coli*	Ec1	ATCC25922	≤0.03	>32	>64	>64	>64	>64	>64	>64	>64
**16**	*P. aeruginosa*	Pa1	ATCC 27853	0,25	>32	>64	>64	>64	>64	>64	>64	>64

Cipro: Ciprofloxacin; Lin: Linezolid.

#### 2.2.2. Antiviral Activity and Cytotoxicity

Antiviral activities of the synthesized compounds were screened against two types of viruses in human epithelial (HeLa) cells for HRV and Human hepatocarcinoma (Huh) cells for HCV. For each compound, the 50% and 90% effective concentration (EC_50_, EC_90_) and the minimal toxic concentration (MTC) or the 50% cytotoxic concentration (CC50) was obtained. None of the compounds exhibited specific antiviral activity, which means that they did not inhibit the replication (induction of viral cytopathogenicity) of any of the viruses tested.

## 3. Experimental

### 3.1. General

NMR spectra were recorded at 250 MHz and 300 MHz (^1^H, ^13^C) Bruker in (DMSO-d_6_, CDCl_3_)using TMS as an internal reference. All chemical shifts (δ) are expressed in parts per million (s, singlet; d, doublet; t, triplet; and m, multiplet) and coupling constants (*J*) are given in Hertz; T (1,2,3-triazole) and B (heterocyclic base). Mass spectra were obtained by using MALDI-TOF and (FAB^+^). Reactions were performed in a domestic microwave oven Model AVM510/WP/WH. DMF was distilled prior to use and stored over molecular sieves 4A. Precoated Merck Silica Gel 60F-254 plates were used for thin layer chromatography (TLC) and the spots were detected under UV light (254 nm). Column chromatography (CLC) was performed using silica gel (0.063–0.2 mm) Fluka. All reagents used were purchased from Aldrich. MICs were determined based on CLSI methodology [37] by a 2-fold broth dilution technique in Mueller Hinton (MH, pH 7.4 Biorad). For *S.pneumoniae* the medium was Brain Heart Infusion broth + 4% red blood cell extract. For *H*. *influenzae* the medium was HTM (Haemophilus Test Medium consisting of MH + 5 g/L yeast extract + hemin 15 mg/L + NAD 20 mg/L). Overnight cultures were diluted to obtain the final inoculum of 105 cfu/well. Incubation was 37 °C overnight in ambient air. 4-Bromobutylacetate (**6**) and 4-azidobutylacetate (**7**) were prepared as described below.

*4-Bromobutylacetate* (**6**). To distilled acetyl bromide (100 mmol, 12.3 g) was added tetrahydrofuran (100 mmol, 7.2 g) dropwise while agitating and cooling with an ice bath. The reaction is fast and exothermic. After addition, the reaction is agitated further during 30 min at room temperature and afterwards the reaction mixture was distilled under reduced pressure. Boiling Point: 92–93 °C (12 mmHg) (96%), ^1^H-NMR (CDCl_3_, δ): 1.6 (m, 4H, CH_2_CH_2_); 2.0 (s, 3H, CH_3_COO); 3.48 (t, 2H, CH_2_Br); 4.03 (t, 2H, OCH_2_).

*4-Azidobutylacetate* (**7**). To a solution of 4-bromobutylacetate (**6**, 10 mmol, 2 g) in anhydrous DMF (60 mL) was added sodium azide (NaN_3_, 15 mmol, 0.9 g). The mixture was brought up to a temperature of 90–95 °C during 4 h. After cooling, the solution was extracted with ether (2 × 50 mL) then washed with brine, and dried (MgSO_4_). After removal of the solvents under reduced pressure, the residual oil was purified on a silica gel column with hexane (91%). ^1^H-NMR (CDCl_3_, δ): 1.64 (m, 4H, CH_2_CH_2_); 2.0 (s, 3H, CH_3_COO); 3.3 (t, 2H, OCH_2_); 4.1 (t, 2H, CH_2_N_3_).

### 3.2. General Procedure for the Synthesis of Monopropargyl Heterocyclic Bases

The mixture of heterocyclic base (thymine, uracil, 6-azauracil and adenine, 1 mmol), K_2_CO_3_ (0.5 mmol) and propargyl bromide (1 mmol) in anhydrous DMF (20 mL) was stirred at room temperature during 24 h. After removal of the solvent under reduced pressure the residue obtained was purified on a silica gel column eluted with CH_2_Cl_2_ and MeOH (99/1).

*N-1-propargyl-6-azauracil* (**9c**). Yield: 55%. ^1^H-NMR (DMSO-d_6_, δ): 3.91 (t, 1H, CH); 4.45 (d, 2H, CH_2_N); 7.46 (s, 1H, H-5); 11.44 (s, 1H, NH). ^13^C-NMR (DMSO-d_6_, δ): 28.37; 73.11; 77.53; 134.61; 148.36; 155.41. FAB-MS, *m/z* calcd for C_6_H_5_N_3_O_2_ (M+H)^+^ 152.04 found,152.

### 3.3. General Procedure for the Synthesis of the N-1, N-3-Bis-propargylpyrimidines/as-Triazines

The mixture of the heterocyclic base (*N*-1-propargyluracil, *N*-1-propargylthymine, and *N*-1-propargyl-6-azauracil, 1 mmol), K_2_CO_3_ (0.5 mmol) and propargylbromide (1.1 mmol) in anhydrous DMF (20 mL) was stirred at room temperature during 15 h. After removal of the solvent under reduced pressure and purification on silica gel column chromatography, we obtained the desired pure product.

*N-1,N-3-dipropargyl-6-azauracil* (**11c**). Yield: 85%. ^1^H-NMR (DMSO-d_6_, δ): 2.98 (t, 1H, CH); 3.44 (t, 1H, CH); 4.48 (d, 2H, CH_2_N); 4.67 (d, 2H, CH_2_N); 7.58 (s, 1H, H-5). ^13^C-NMR (DMSO-d_6_, δ): 29.38; 39.40; 73.50; 75.43; 77.36; 77.64; 134.81; 147.11; 154.90. FAB-MS, *m/z* calcd for C_8_H_8_N_2_O_2 _(M+H)^+^ 190.05 found, 190.

### 3.4. General Procedure for the Synthesis of the Triazole acyclonucleoside Derivatives

The mixture of alkylazide (5 mmol), Et_3_N (1 mmol), *N*-propargylbase (1 mmol) and CuI (0.1 mmol) was irradiated in the microwave oven at power level (300 W) for 1 min without solvent. K_2_CO_3_ (2 mmol) in methanol (10 mL) was added directly to reaction mixture. The mixture was stirred for additional 3 h at room temperature (or in 30 mL of methanol saturated with ammonia at 0 °C during 24 h). When TLC analysis showed no starting material, solvent was removed under reduced pressure, and the residue was purified on silica gel eluting with dichloromethane and methanol.

*1-[[1-[(4-Hydroxybutyl)methyl]-1,2,3-triazol-4-yl]methyl]uracil* (**10a**). Yield: 91%. ^1^H-NMR (DMSO-d_6_, δ): 1.44–1.29 (m, 2H, CH_2_); 1.90–1.75 (m, 2H, CH_2_); 3.45–3.37 (m, 2H, OCH_2_); 4.35 (t, 2H, CH_2_-T, *J =* 7.13); 4.47 (t, 1H, OH, *J =* 4.62); 4.94 (s, 2H, T-CH_2_-B); 5.60 (d, 1H, H-5, *J =* 7.85 Hz); 7.75 (d, 1H, H-6, *J =* 7.87 Hz); 8.08 [s, 1H, H-5(triazole)]; 11.31 (s, 1H, NH). ^13^C-NMR (DMSO-d_6_, δ): 26.56; 29.24; 42.39; 49.34; 59.98; 101.23; 123.36; 142.23; 145.44; 150.72; 163.65. (MALDI-TOF-MS) *m/z* calcd for C_11_H_15_N_5_O_3_ 265.12, found, 266.87.

*1-[[1-[(4-Hydroxybutyl)methyl]-1,2,3-triazol-4-yl]methyl]thymine* (**10b**). Yield: 92%. ^1^H-NMR (DMSO-d_6_, δ): 1.44–1.30 (m, 2H, CH_2_); 1.76 (s, 3H, CH_3_); 1.85 (m, 2H, CH_2_); 3.44–3.36 (m, 2H, OCH_2_); 4.34 (t, 2H, CH_2_-T, *J =* 7.15 Hz); 4.47 (t, 1H, OH, *J =* 5.10 Hz); 4.90 (s, 2H, T-CH_2_-B); 7.62 (s, 1H, H-6), 8.07 [s, 1H, H-5(triazole)]; 11.30 (s, 1H, NH). ^13^C-NMR (DMSO-d_6_, δ): 11.91; 26.56; 29.25; 42.23; 49.33; 59.98; 108.83; 123.32; 141.11; 142.40; 150.69; 164.24. (MALDI-TOF-MS) *m/z* calcd for C_12_H_17_N_5_O_3_ 279.13, found, 279.07.

*1-[[1-[(4-Hydroxybutyl)methyl]-1,2,3-triazol-4-yl]methyl]-6-azauracil* (**10c**). Yield: 90%. ^1^H-NMR (DMSO-d_6_, δ): 1.37 (m, 2H, CH_2_); 1.90–1.73 (m, 2H, CH_2_); 3.43–3.37 (m, 2H, OCH_2_); 4.32 (t, 2H, CH_2_-T, *J =* 7.16); 4.47 (s, 1H, OH); 4.98 (s, 2H, T-CH_2_-B); 7.56 (s, 1H, H-5); 8.02 [s, 1H, H-5(triazole)]; 12.64 (s, 1H, NH). ^13^C-NMR (DMSO-d_6_, δ): 26.55; 29.25; 34.52; 49.27; 59.99; 123.32; 134.75; 141.48; 148.86; 155.89. (MALDI-TOF-MS) *m/z* calcd for C_10_H_14_N_6_O_3_ 266.11, found, 266.64.

*1-[[1-[(4-Hydroxybutyl)methyl]-1,2,3-triazol-4-yl]methyl]adenine* (**10d**). Yield: 89%. ^1^H-NMR (DMSO-d_6_, δ): 1.35 (m, 2H, CH_2_); 1.79 (m, 2H, CH_2_); 3.39 (m, 2H, OCH_2_); 3,47 (s, 1H, OH); 4.33 (t, 2H, T, *J =* 7.13 Hz); 5.44 (s, 2H, T-CH_2_-B); 7.24 (s, 2H, NH_2_); 8.10 [s, 1H, H-5(triazole)]; 8.15 and 8.20 (s, 2H, H-2 and H-8). ^13^C-NMR (DMSO-d_6_, δ): 26.55; 29.21; 38.02; 49.33; 59.95; 118.57; 123.39; 140.61; 142.42; 149.29; 155.96; 152.55. (MALDI-TOF-MS) *m/z* calcd for C_12_H_16_N_8_O 288.14, found, 288.11.

*1,3-bis-[[1-[(4-Hydroxybutyl)methyl]-1,2,3-triazol-4-yl]methyl]uracil* (**12a**). Yield: 90%. ^1^H-NMR (DMSO-d_6_, δ): 1.44–1.29 [m, 4H, 2 × (CH_2_)]; 1.90–1.73 [m, 4H, 2 × (CH_2_)]; 3.40 [m, 4H, 2 × (OCH_2_)]; 4.32 [m, 4H, 2 × (CH_2_)]; 4.47 [s, 2H, 2 × (OH)]; 5.02 [s, 4H, 2 × (T-CH_2_-B)]; 5.78 (d, 1H, H-5, *J =* 7.89 Hz); 7.84 (d, 1H, H-6, *J =* 7.91 Hz); 7.92 [s, 1H, H-5(triazole)]; 8.10 [s, 1H, H-5(triazole)]. ^13^C-NMR (DMSO-d_6_, δ): 2 × (C) 26.56; 2 × (C) 29.24; 35.84; 43.59; 49.22; 49.36; 2 × (C) 59.99; 100.50; 123.15; 123.52; 141.98; 142.48; 144.21; 150.74; 161.99. (MALDI-TOF-MS) *m/z* calcd for C_18_H_26_N_8_O_4_ 418.21, found, 418.46

*1,3-bis-[[1-[(4-Hydroxybutyl)methyl]-1,2,3-triazol-4-yl]methyl]thymine* (**12b**). Yield: 92%. ^1^H-NMR (DMSO-d_6_, δ): 1.45–1.30 [m, 4H, 2 × (CH_2_)]; 1.90–1.75 [m, 7H, 2 × (CH_2_) and CH_3_]; 3.40 [m, 4H, 2 × (OCH_2_)]; 4.32 [m, 4H, 2 × (CH_2_-T)]; 4.49 [s, 2H, 2 × (OH)]; 4.98 (s, 2H, T-CH_2_-B); 5.05 (s, 2H, T-CH_2_-B); 5.74 (s, 1H, H-6); 7.91 [s, 1H, H-5(triazole)]; 8.09 [s, 1H, H-5(triazole)]. ^13^C-NMR (DMSO-d_6_, δ): 12.56; 2 × (C) 26.57; 2 × (C) 29.26; 36.10; 43.43; 49.21; 49.35; 2 × (C) 59.98; 108.09; 123.16; 123.47; 140.05; 142.15; 142.56; 150.58; 162.73. (MALDI-TOF-MS) *m/z* calcd for C_19_H_28_N_8_O_4_ 432.22, found, 432.64.

*1,3-bis-[[1-[(4-Hydroxybutyl)methyl]-1,2,3-triazol-4-yl]methyl]-6-azauracil* (**12c**). Yield: 91%. ^1^H-NMR (DMSO-d_6_, δ): 1.36 [m, 4H, 2 × (CH_2_)]; 1.81 [m, 4H, 2 × (CH_2_)]; 3.37 [m, 4H, 2 × (OCH_2_)], 4.32 [m, 4H, 2 × (CH_2_-T)]; 4.43 (s, 2H, 2 × (OH); 4.80 (s, 2H, T-CH_2_-B); 5.06 (s, 2H, T-CH_2_-B); 7.14 (s, 1H, H-5); 7.91 [s, 1H, H-5(triazole)]; 7.98 [s, 1H, H-5(triazole)]. ^13^C-NMR (DMSO-d_6_, δ): 2 × (C) 26.5; 2 × (C) 29.27; 35.71; 36.65; 2 × (C) 49.23; 2 × (C) 59.95; 122.71; 2 × (C) 122.94; 2 × (C) 141.89; 145.40; 155.46. (MALDI-TOF-MS) *m/z* calcd for C_17_H_25_N_9_O_4_ 419.20, found, 419.17.

## 4. Conclusions

A series of triazole carboacyclonucleosides with various nucleobase moieties appended on the triazole were synthesized efficiently using a convenient one-step click azide-alkyne cycloaddition reaction under solvent-free microwave irradiation. All compounds synthesized were evaluated for their antibacterial and antiviral activities but none exhibited specific activity so far. Further applications of the click azide-alkyne cycloaddition process are currently under investigation and will be reported in due course.
